# Contact Force-Sensing versus Standard Catheters in Non-Fluoroscopic Radiofrequency Catheter Ablation of Idiopathic Outflow Tract Ventricular Arrhythmias

**DOI:** 10.3390/jcm11030593

**Published:** 2022-01-25

**Authors:** Grzegorz Karkowski, Marcin Kuniewicz, Andrzej Ząbek, Edward Koźluk, Maciej Dębski, Paweł T. Matusik, Jacek Lelakowski

**Affiliations:** 1 Department of Electrocardiology, The John Paul II Hospital, 31-202 Kraków, Poland; gkarkowski@interia.pl (G.K.); kuniewiczm@gmail.com (M.K.); andrzej_j_z@poczta.onet.pl (A.Z.); jacek.lelakowski@uj.edu.pl (J.L.); 2 Department of Anatomy, Jagiellonian University Medical College, 31-008 Kraków, Poland; 3 Department of Cardiology, Medical University of Warsaw, 02-097 Warsaw, Poland; ekozluk@vp.pl; 4 Department of Cardiology, Norfolk and Norwich University Hospital, University of East Anglia, Norwich NR4 7TJ, UK; maciekdebski@gmail.com; 5 Department of Electrocardiology, Institute of Cardiology, Jagiellonian University Medical College, 31-008 Kraków, Poland

**Keywords:** contact force, non-fluoroscopic ablation, outflow tracts, premature ventricular contractions, ventricular arrhythmias

## Abstract

Background: Adequate contact between the catheter tip and tissue is important for optimal lesion formation and, in some procedures, it has been associated with improved effectiveness and safety. We evaluated the potential benefits of contact force-sensing (CFS) catheters during non-fluoroscopic radiofrequency catheter ablation (NF-RFCA) of idiopathic ventricular arrhythmias (VAs) originating from outflow tracts (OTs). Methods: A group of 102 patients who underwent NF-RFCA (CARTO, Biosense Webster Inc., Irvine, CA, USA) of VAs from OTs between 2014 to 2018 was retrospectively analyzed. Results: We included 52 (50.9%) patients in whom NF-RFCA was performed using CFS catheters and 50 (49.1%) who were ablated using standard catheters. Arrhythmias were localized in the right and left OT in 70 (68.6%) and 32 (31.4%) patients, respectively. The RFCA acute success rate was 96.1% *(n* = 98) and long-term success during a minimum 12-month follow-up (mean 51.3 ± 21.6 months) was 85.3% (*n* = 87), with no difference between CFS and standard catheters. There was no difference in complications rate between CFS (*n* = 1) and standard catheter (*n* = 2) ablations. Conclusions: There is no additional advantage of CFS catheters use over standard catheters during NF-RFCA of OT-VAs in terms of procedural effectiveness and safety.

## 1. Introduction

The non-fluoroscopic (NF) radiofrequency catheter ablation (RFCA) of idiopathic ventricular arrhythmias (VAs) from outflow tracts (OTs) has a good effectiveness and safety profile, but arrhythmia recurrence is not uncommon and may be observed in 4–12.5% of the patients [1,2,3,4,5]. Activation mapping of ventricular OTs arrhythmias reveals focal origin in most cases. Besides precise mapping, procedural success depends on good ablation lesion formation. Multiple factors influence the optimal creation of lesions, some of which are operator-dependent and include the type of energy source (e.g., cryoablation, radiofrequency current), power setting, application duration, catheter tip orientation or catheter type and size. Importantly, adequate contact between the catheter tip and tissue plays an essential role in lesion formation [3,6,7]. Inadequate contact force (CF) will decrease lesion size and reduce ablation effectiveness [8]. On the other hand, excessive CF might lead to mechanical injury or tissue overheating, causing steam pop, which is a life-threatening complication [9,10]. Traditionally, evaluation of optimal CF has been based on operator-dependent parameters such as tactile feedback, visual assessment of catheter motion and physical parameters such as impedance drop, tip temperature value or electrogram amplitude change during application. Unfortunately, those parameters have a poor correlation with real CF during application and have a limited role in clinical practice [6,8]. Introduction and validation of contact force-sensing catheters (CFS) capable of real-time catheter tip–tissue contact force measurement have helped optimize lesion formation and increase procedural safety [11,12]. The benefits of CFS catheters have been previously proven in atrial fibrillation (AF) RFCA [11,12,13]. Due to outflow tract anatomy and ventricular tissue thickness, especially in septal location, such as left ventricular (LV) summit, optimal CF is theoretically very desirable, especially in the NF procedure. However, published data are contradictory and recent studies revealed no advantage of CFS guidance in fluoroscopic RFCA compared to standard open irrigated catheters of OT-VAs [14,15]. Due to the increasing role of NF-RFCA, we aimed to evaluate the potential benefits of open irrigated CFS catheters in RFCA of idiopathic VAs from OTs without fluoroscopy guidance.

## 2. Materials and Methods

### 2.1. Patients

We retrospectively analyzed a group of 102 patients who between 2014 and 2018 underwent NF-RFCA of premature ventricular contractions (PVC) from OTs. Procedures were performed or supervised by operators experienced in NF and fluoroscopic RFCA. NF-RFCA was defined as ablation performed without use of fluoroscopy (zero-fluoroscopy). The choice of catheter type was left to the decision of the operator. Patients listed for RFCA had idiopathic, symptomatic VAs with high daily PVC burden (generally minimum 10% PVC per day) from the right or left ventricular OT (RVOT or LVOT), including arrhythmias originating from aortic cusps. Antiarrhythmic medications were suspended 48 h before the ablation. None of the patients was on amiodarone or sotalol before ablation. The CFS catheters were used for mapping and ablation in 52 patients, while standard catheters were without CFS in the remaining 50 patients. All patients provided written consent to undergo the procedure. The local ethics committee waived the need for their opinion to perform the study due to the retrospective character of the project (L.dz.OIL/KBL/82/2021). The study protocol conformed to the ethical guidelines of the 1975 Declaration of Helsinki.

### 2.2. Mapping and Ablation Protocol

**Figure 1:**
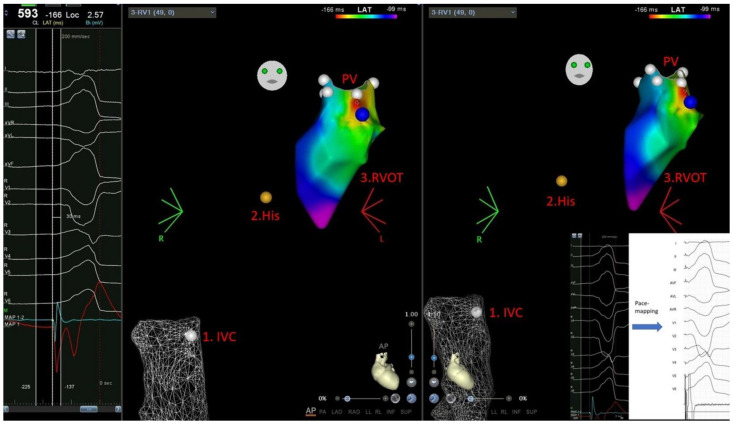
A. Activation map of premature ventricular contraction (PVC) from right ventricular outflow tract-RVOT (CARTO Biosense Webster Inc., Irvine, CA, USA) performed without fluoroscopy. First stage of the procedure is FAM of IVC and CF sensor calibration (if used). Second stage is His potential (yellow dot) localization and performance of respiratory gating. The third stage is performance of point-by-point activation mapping supported with pace-mapping. In showed case, earliest endocardial potential advancing PVC-QRS-30 ms (with automatic reference annotation)–spot of RF application, blue dot-a spot of optimal pace-mapping with compatibility with PVC > 95%. White dots–PV marked. AP and RAO projections. Abbreviations: CF, contact force; FAM, fast anatomical mapping; IVC, inferior vena cava; PVC, premature ventricular contraction; PV, pulmonary valve.

All ablations were guided by CARTO electroanatomic mapping system (Biosense Webster Inc., Diamond Bar, CA, USA). In standard catheter group, 3.5 mm open irrigated tip catheters (Biosense Webster Navistar Thermocool) were used, while in the remaining patients 3.5 mm open irrigated-tip catheters with CFS (Thermocool SmartTouch^®^, Biosense Webster, Irvine, CA, USA) were used. The setting of RF application parameters: energy in RVOT 30–40 Wats (W), in LVOT 20–30 W, the flow of irrigation: of 15–30 mL/min, time of application (60 s), temperature limits (max 45 °C) were the same in both groups. RF applications were performed at the CF target in the CFS group, i.e., between 10–30 g. To determine the PVC origin, activation mapping was used to look for the earliest endocardial potential advancing QRS during PVC. Additionally, in the earliest activation spot, pace-mapping was applied to confirm PVC localization with PVC compatibility of at least 95% of the complexes analyzed by electrophysiological recording system (LABSYSTEM™ PRO, Boston Scientific, Marlborough, MA, USA) with as low as possible pacing output (1–2 mV/0.5 ms over pacing capture loss, max. pacing output 12 mV/0.5 ms), Figure 1.

The technique of NF-RFCA was described previously [5]. CFS catheters were calibrated to set the baseline value before mapping and ablation in inferior vena cava (1–2 cm below right atrium) or in descending aorta (1–2 cm below aortic arch), depending on venous or arterial approach.

During the RFCA of RVOT-VAs, a single or double (for coronary sinus diagnostic electrode) femoral vein puncture was performed. In left-sided arrhythmias, single femoral artery access was obtained. In some cases, both right and left access was used. The procedure was performed under local anesthesia or conscious sedation, if necessary.

The acute (short-term) efficacy of RFCA was defined as no recurrent PVCs after 15 min from the last RF application. The patients were challenged with isoproterenol only in those cases where PVCs required pharmacological induction before ablation. A minimum 12-month follow-up period was required. Long-term efficacy was defined as a significant arrhythmia reduction (over 80% reduction of arrhythmia burden) after at least three months in repeated 24-h ECG monitoring (every 6–12 months). Antiarrhythmic medication was not continued after ablation, except beta-blockers or calcium blockers if used for another indication, i.e., hypertension. Antiarrhythmics were introduced in the case of symptomatic arrhythmia recurrence. All necessary medical follow-up data (12 lead ECG, 24-h Holter ECG, repeat ablation information) were obtained from outpatient medical records.

### 2.3. Twenty-Four-Hour Holter Monitoring and Echocardiography

Twenty-four-hour Holter ECG monitoring was performed before ablation, three months after RF ablation, and at least once a year after this period. ECG recordings were performed according to the Polish Cardiac Society Guidelines [16].

Transthoracic echocardiography (TTE) was performed in all patients. Exams were done with Vivid S6 (GE Healthcare, Chicago, IL, USA) device, according to the European Association for Cardiovascular Imaging (EACVI) and Polish guidelines at the time of patient enrollment [17].

### 2.4. Statistical Analysis

Continuous variables are presented as means and standard deviations (SD) or medians and interquartile ranges, as appropriate, and were compared using the Student’s *t*-test or the Mann–Whitney U test. Categorical variables are expressed as absolute numbers and frequencies and were compared with the chi-square test, including Yates’ correction for continuity or Fisher’s exact test. The graphic presentation of the long-term premature ventricular contraction ablation success is shown using Kaplan–Meier curves, which were compared using the log-rank test. All statistical tests were 2-tailed, and a *p* < 0.05 was considered statistically significant. Data were analyzed using IBM SPSS Statistics Version 25.0 software (IBM Corp, Armonk, NY, USA).

## 3. Results

### 3.1. Baseline Patients’ Characteristic

**Figure 2:**
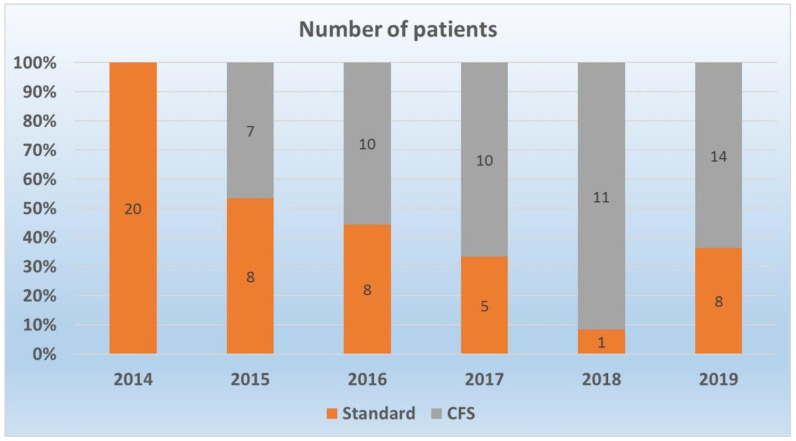
Distribution of ablation catheters type over time. Abbreviations: CFS—contact force-sensing.

The study included 102 patients, 52 (50.9%) had CFS guided RFCA and 50 (49.1%) RFCA using standard catheters. In 2014, only standard catheters were used for RFCA, but from 2016, CFS catheters started to be used more often than standard ones. The distribution of used catheter types during the years is presented in Figure 2.

The mean age of the patients was 43.4 years (SD 14.8); 63 (61.8%) were female. PVCs were originating from RVOT and LVOT in 70 (68.6%) and 32 (31.4%) patients, respectively. In 17 cases (16.7%), the procedure was performed as a re-do ablation (5 cases (10.0%) in standard group and 12 cases (23.1%) in CFS group, *p* = 0.132). The patients’ age, comorbidities such as hypertension, coronary artery disease, and diabetes mellitus, and history of AF and medical therapy at baseline and during follow-up were similar in CFS and standard catheter groups. Detailed patients’ baseline characteristics are presented in Table 1.

### 3.2. Procedural Characteristic and Ablation Effectiveness

The median procedural time was 85 min (Q1–Q3 65.0–100.7 min) and was significantly longer in the CFS group (90.0 min, Q1–Q3 70.0–120.0 min) compared with the standard catheter group (80.0 min, Q1–Q3 65.0–90.0 min) (*p* = 0.029). Notably, neither the site of the procedure (RVOT/LVOT) nor procedural time varied significantly between groups (Table 2).

**Figure 3:**
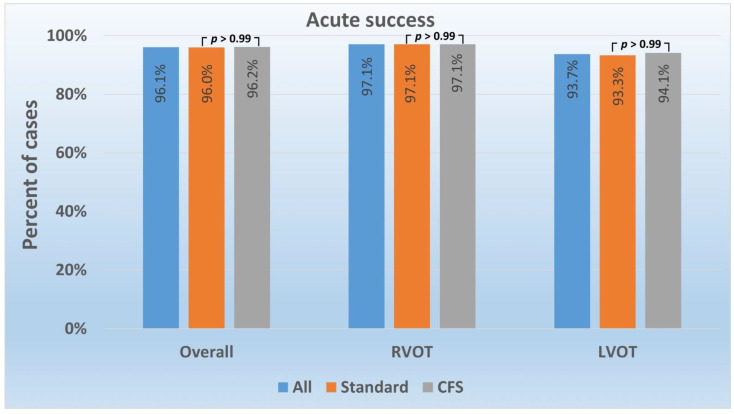
Premature ventricular contraction ablation acute success according to catheter type and arrhythmia localization. Abbreviations: CFS—contact force-sensing; LVOT—left ventricular outflow tract; RVOT—right ventricular outflow tract.

The overall acute success was 96.1% (*n* = 98), and it did not differ between CFS (96.2%) and standard (96.0%) RFCA (*p* > 0.99) in the whole group and in patients with RVOT or LVOT arrhythmia origin considered separately (Table 2 and Figure 3).

**Figure 4:**
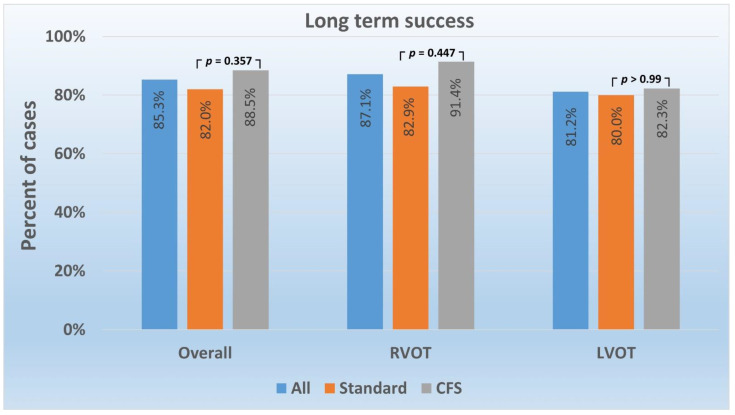
Premature ventricular contraction ablation long-term success according to catheter type and arrhythmia localization. Abbreviations: CFS—contact force-sensing; LVOT—left ventricular outflow tract; RVOT—right ventricular outflow tract.

In the follow-up of 51.3 months (SD 21.6 months), long-term success was achieved in 85.3% (*n* = 87) of patients, and there was no difference between patients ablated using CFS (88.5%) and standard (82.0%) catheters (*p* = 0.357). The long-term success of RFCA was similar in CFS and the standard catheter group and did not depend on LVOT or RVOT PVCs origin (Figure 4).

**Figure 5:**
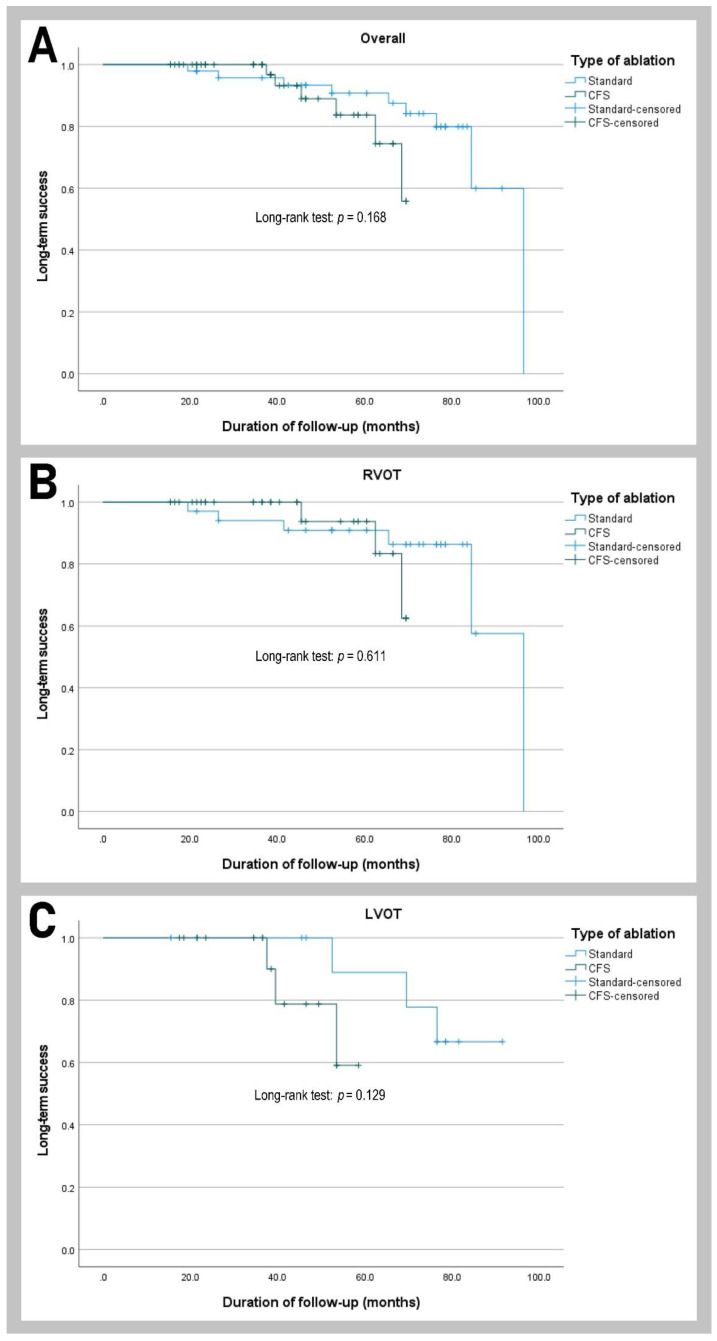
Kaplan–Meier curves of the long-term ablation success of the premature ventricular contractions according to catheter type and arrhythmia localization. (**A**) overall, (**B**) RVOT group, (**C**) LVOT group. Abbreviations: CFS—contact force-sensing; LVOT—left ventricular outflow tract; RVOT—right ventricular outflow tract.

In addition, there was no significant difference in long-term success between standard and CFS catheters (Figure 5).

However, the median follow-up was significantly shorter in the CFS group than in the standard catheter group (40.0 months vs. 69.5 months, *p* < 0.001). Detailed results are presented in Table 2.

### 3.3. Complications

There was no difference in complication rate between CFS and standard catheter ablations (*p* > 0.99). However, there were three (2.9%) major perioperative complications: one pseudoaneurysm after femoral artery access in standard catheters group (treated by thrombin injection) and two patients developed pericarditis in CFS catheters group. In one case after ablation in the aortic root and in the second case after ablation in the tricuspid valve region. In one case, pericarditis required pharmacotherapy. In the second case, despite medication use (ibuprofen and colchicine), the patient required pericardiocentesis two weeks later due to the symptomatic pericardial effusion (sub-acute perforation could not be excluded).

## 4. Discussion

To the best of our knowledge, this is the first study evaluating the role of CFS catheters in RFCA OT-VAs without fluoroscopy. Recent publications have demonstrated the safety and efficacy of NF-RFCA in OT arrhythmias, but the role of CFS catheters in this setting is unknown [1,4,5]. The results of our study show no advantages of CFS over standard catheters in NF-RFCA of OT-VAs, in keeping with previous publications concerning the same arrhythmia type but performed with fluoroscopy guidance [14,15,18]. Reichlin et al. [14] and Abraham et al. [15] compared the effectiveness and safety of CFS vs. standard catheters and showed similar outcomes in LVOT and RVOT arrhythmia ablation. In other studies, the use of CFS catheters only shortened a procedural, fluoroscopy and ablation time [18]. Conversely, we observed a longer procedural time in the CFS guidance group which might be explained by often time-consuming attempts to achieve intended CF (>10 g) with relatively stiffer CFS catheters compared to standard ones and also time needed for CFS catheters calibration, which should be done after CFS catheter introduction and every time when the operator makes a decision to map and/or ablate another ventricle during NF-RFCA of OT-VAs. Nevertheless, the lack of apparent benefit of CFS catheters in OT-VAs seems surprising if we consider the well-studied advantages of CFS catheters in RFCA of AF. TOCCATA [19] was the first study that showed the value of optimal CF on pulmonary vein isolation effectiveness. All patients treated with an average CF of <10 g experienced AF recurrences, unlike patients with CF > 20 g who were free of AF in 12-month follow-up. Furthermore, TOCCASTAR, a prospective randomized multicenter trial, confirmed the usefulness of CFS catheters and the correlation between optimal CF and pulmonary vein isolation effectiveness. Notably, the improved procedural outcomes were evident when >90% of applications were in the optimal range with a minimum of 10 g. For VA ablation there is lack of such strong data indicating a clear CF cut off needed for procedural outcome improvement. In a recent retrospective study by Abraham et al. [15] (CFS catheters *n* = 75 vs. standard catheters *n* = 75), the median CF was 12.0 (9.5–18.5) g and did not differ significantly between acute success and failure. Larger studies regarding optimal CF in VAs ablation would be valuable. Besides CF value, used CFS catheters provide information on catheter tip direction and angle in relation to cardiac tissue (pointing vector). Standard catheters, used in the current study, show only the information on tip direction relative to the operator coded by color of the catheter, which is less intuitive and clear.

Acute and long-term pulmonary vein isolation efficiency depends mainly on durable, transmural and continuous (dense) ablation lines in the left atrium, while a fundamental goal is an optimal lesion formation for RF ablation. Distinctly for idiopathic OT-VAs, lesion transmurality is not usually necessary for ablation success. Moreover, it is nearly impossible to achieve it even with a high CF. Additionally, a curvier catheter route to RVOT or LVOT and, connected with it, more difficult mapping process compared to transseptal (straight) left atrium access might favor stiffer CFS catheters in AF ablations. Based on the present findings and recently published data, we speculate that the most critical step in idiopathic OT-VAs is a very detailed mapping process. It appears that CFS guidance impacts neither this process nor RF application efficiency to a significant degree. However, in a case of questionable PVC origin (RVOT or LVOT), information of appropriate CF during unsuccessful RF application might be useful, pointing towards the necessity for further mapping in other localizations (another OT) or use of an epicardial approach due to deep (from the endocardium) PVC origin. The epicardial region is mainly reached via the venous system using a coronary sinus. Deep arrhythmia foci rarely require ablation from the left and right OTs or with simultaneous bipolar ablation. In contrast, during substrate mapping in patients with structural heart disease, point acquisition requires sufficient CF to create an adequate voltage map without false low-voltage zones [20]. The minimum value of optimal CF during the systolic/diastolic phase (to obtain contact) has been estimated at 9 g and 8 g for RV and LV, respectively [20].

The CFS technology should also provide safety during RFCA. In NF-RFCA the operator relies only on catheter visualization in 3 D mapping systems and information of real-time CF might be potentially very important. Excessive CF during mapping and ablation might produce cardiac or vessel injury leading to massive bleeding or tamponade. Those major complications are rare but potentially life-threatening. Real-time CF measurement gives the operators (especially those inexperienced) confidence throughout the process of catheter insertion, mapping and RF application, mainly when RFCA is performed without fluoroscopy guidance, but we showed that it did not impact on procedural safety. Standard catheters are less stiff and more steerable with the right level the CF information. In an ex vivo swine model study, Shah et al. [21] defined lower minimum perforating forces in the right and left ventricle, at 159 g and 227 g, respectively. Those values were lower in previously ablated tissue, and, additionally, time to perforation was shorter when catheters were introduced by a steerable long sheath [21]. Otherwise, in VAs ablations, increasing CF and power during RF application might cause steam pops and lead to cardiac perforation, embolic stroke and ventricular septal defect, which have been reported to be more likely than mechanical perforation [22,23].

According to Akca et al.’s meta-analysis concerning different types of ablations, the impact of CFS catheters on procedural safety was demonstrated only in AF [24]. Additionally, in the TOCCATA study, cardiac perforation was linked to excessive CF based on blinded CFS measurements [19]. On the contrary, in previously published data and our study results, there is no evidence of the significant implication of the CFS catheter on safety during ventricular RFCA, independent of the presence of structural heart disease, arrhythmia localization, or fluoroscopy use [14,15,18,24,25,26]. Novel techniques are introduced in cardiac electrophysiology [27]. The use of non-fluoroscopic imaging during catheter ablation is more frequently used than before [28,29]. It may be speculated that CFS catheters might be of special value in this setting in patients with congenital heart disease, while in the case of ventricular arrhythmias originating from left ventricle, there may be limited additional benefit in patients with left ventricular hypertrophy.

### Limitations of the Study

The primary study limitation is a relatively small patient group (especially those with LVOT PVCs origin). There was a trend towards higher success rate using CFS catheters during RVOT-VA ablations, which was not statistically significant probably due to a relatively small group of investigated patients. The study had retrospective design and there was no randomization of ablations using CFS catheters and standard catheters. Furthermore, an important limitation of this study was that the decision of using CFS or standard catheters was based on the choice of each operator. The proportion of CFS catheters has generally increased during recent years and is not equal to standard catheters in particular years. Additionally, there is no information on CF during applications but only the targeted value of optimal contact and the number of RF applications in both groups (power and time settings were the same).

## 5. Conclusions

The results of our study show no additional advantages of CFS catheters during NF-RFCA of OT-VAs compared to standard catheters regarding procedural effectiveness and safety.

## Figures and Tables

**Table 1:** Patient characteristics.

Variable	Total (*n* = 102)	Standard Catheter Ablation (*n* = 50)	CFS Catheter Ablation (*n* = 52)	*p*-Value
Age (years)	42.0 (32.7–55.0)	42.0 (32.7–53.5)	42.0 (32.2–55.7)	*p* = 0.987
Female, *n* (%)	63 (61.8)	29 (58.0)	34 (65.4)	*p* = 0.443
RVOT PVCs origin, *n* (%)	70 (68.6)	35 (70)	35 (67.3)	*p* = 0.770
LVOT PVCs origin, *n* (%)	32 (31.4)	15 (30)	17 (32.7)	
Hypertension, *n* (%)	27 (26.5)	13 (26.0)	14 (26.9)	*p* = 0.916
History of CAD, *n* (%)	12 (11.8)	6 (12.0)	6 (11.5)	*p* = 0.942
Diabetes mellitus, *n* (%)	5 (4.9)	4 (8.0)	1 (1.9)	*p* = 0.200
Presence of CIED, *n* (%)	2 (2.0)	2 (4.0)	0 (0.0)	*p* = 0.238
History of AF, *n* (%)	2 (2.0)	0 (0.0)	2 (3.8)	*p* = 0.495
Invasive correction of atrial septal defect *, *n* (%)	2 (2.0)	1 (2.0)	1 (1.9)	*p* > 0.99
Beta blocker, *n* (%)	26 (25.5)	12 (24.0)	14 (26.9)	*p* = 0.735
Calcium-channel blocker **, *n* (%)	3 (2.9)	2 (4.0)	1 (1.9)	*p* = 0.614
Propafenone, *n* (%)	11 (10.8)	7 (14.0)	4 (7.7)	*p* = 0.305
Number of antiarrhythmic drugs after ablation	0.0 (0.0–1.0)	0.0 (0.0–1.0)	0.0 (0.0–1.0)	*p* = 0.605

Data are presented as median (Q1–Q3) or number (percentage). Abbreviations: AF—atrial fibrillation; BMI—body mass index, CAD—coronary artery disease; CIED—cardiac implantable electronic device; CFS—contact force-sensing. * Interatrial septal occluder or surgical correction, ** Verapamil or diltiazem.

**Table 2:** Procedural parameters and complications.

Parameter	Total (*n* = 102)	Standard Catheter Ablation (*n* = 50)	CFS Catheter Ablation (*n* = 52)	*p*-Value
Duration of procedure (min)	85.0 (65.0–100.7)	80.0 (65.0–90.0)	90.0 (70.0–120.0)	*p* = 0.029
Duration of procedure in only RVOT ablation site (min)	80.0 (60.0–106.2)	70.0 (65.0–85.0)	85.0 (60.0–120.0)	*p* = 0.074
Duration of procedure in only LVOT ablation site (min)	90.0 (76.2–100.0)	90.0 (75.0–96.0)	96.0 (76.0–125.0)	*p* = 0.261
Re-ablation at baseline, *n* (%)	17 (16.7)	5 (10)	12 (23.1)	*p* = 0.132
Use of isoproterenol, *n* (%)	21 (20.6)	11 (22.0)	10 (19.2)	*p* = 0.730
Overall acute success, *n* (%)	98 (96.1)	48 (96.0)	50 (96.2)	*p* > 0.99
RVOT acute success, *n* (%)	68 (97.1)	34 (97.1)	34 (97.1)	*p* > 0.99
LVOT acute success, *n* (%)	30 (93.7)	14 (93.3%)	16 (94.1)	*p* > 0.99
Overall long-term success, *n* (%)	87 (85.3)	41 (82.0)	46 (88.5)	*p* = 0.357
RVOT long-term success, *n* (%)	61 (87.1)	29 (82.9)	32 (91.4)	*p* = 0.477
LVOT long-term success, *n* (%)	26 (81.2)	12 (80.0)	14 (82.3)	*p* > 0.99
Complications, *n* (%)	3 (2.9)	2 (4.0)	1 (1.9)	*p* = 0.614
Duration of follow-up (months)	52.5 (34.5–69.5)	69.5 (46.2–77.5)	40.0 (24.0–56.7)	*p* < 0.001

Data are presented as median (Q1–Q3) or number (percentage). Abbreviations: CFS—contact force-sensing; LVOT—left ventricular outflow tract; RVOT—right ventricular outflow tract.

## Data Availability

The authors confirm that the data supporting the findings of this study are available within the article.

## References

[1] Zhu T.Y., Liu S.R., Chen Y.Y., Xie L.Z., He L.W., Meng S.R., Peng J. (2016). Zero-fluoroscopy catheter ablation for idiopathic premature ventricular contractions from the aortic sinus cusp. Nan Fang Yi Ke Da Xue Xue Bao.

[2] Koźluk E., Gawrysiak M., Piątkowska A., Lodziński P., Kiliszek M., Małkowska S., Zaczek R., Piątkowski R., Opolski G., Kozłowski D. (2013). Radiofrequency ablation without the use of fluoroscopy—In what kind of patients is it feasible?. Arch. Med. Sci..

[3] Kozluk E., Rodkiewicz D., Piątkowska A., Opolski G. (2018). Safety and efficacy of cryoablation without the use of fluoroscopy. Cardiol. J..

[4] Styczkiewicz K., Ludwik B., Śledź J., Lipczyńska M., Zaborska B., Kryński T., Deutsch K., Morka A., Kukla P., Styczkiewicz M. (2019). Long-term Follow-Up and Comparison of Techniques in Radiofrequency Ablation of Ventricular Arrhythmias Originating from the Aortic Cusps (AVATAR Registry). Pol. Arch. Intern Med..

[5] Karkowski G., Kuniewicz M., Koźluk E., Chyży T., Ząbek A., Dusza M., Lelakowski J. (2020). Non-fluoroscopic radiofrequency catheter ablation of right and left sided ventricular arrhythmias. Postepy Kardiol. Interwencyjnej.

[6] Zheng X., Walcott G.P., Hall J.A., Rollins D.L., Smith W.M., Kay G.N., Ideker R.E. (2000). Electrode impedance: An indicator of electrode-tissue contact and lesion dimensions during linear ablation. J. Interv. Card. Electrophysiol..

[7] Wittkampf F.H.M., Nakagawa H. (2006). RF catheter ablation: Lessons and lesions. Pacing Clin. Electrophysiol..

[8] Kumar S., Morton J.B., Lee G., Halloran K., Kistler P.M., Kalman J.M. (2015). High incidence of low catheter-tissue contact force at the cavotricuspid isthmus during catheter ablation of atrial flutter: Implications for achieving isthmus block. J. Cardiovasc. Electrophysiol..

[9] Yokoyama K., Nakagawa H., Shah D.C., Lambert H., Leo G., Aeby N., Ikeda A., Pitha J.V., Sharma T., Lazzara R. (2008). Novel Contact Force Sensor Incorporated in Irrigated Radiofrequency Ablation Catheter Predicts Lesion Size and Incidence of Steam Pop and Thrombus. Circ. Arrhythm. Electrophysiol..

[10] Seiler J., Roberts-Thomson K.C., Raymond J.M., Vest J., Delacretaz E., Stevenson W.G. (2008). Steam pops during irrigated radiofrequency ablation: Feasibility of impedance monitoring for prevention. Heart Rhythm.

[11] Sarkozy A., Shah D., Saenen J., Sieira J., Phlips T., Boris W., Namdar M., Vrints C. (2015). Contact force in atrial fibrillation: Role of atrial rhythm and ventricular contractions: Co-Force Atrial Fibrillation Study. Circ. Arrhythm. Electrophysiol..

[12] Lin H., Chen Y.H., Hou J.W., Lu Z.Y., Xiang Y., Li Y.G. (2017). Role of contact force-guided radiofrequency catheter ablation for treatment of atrial fibrillation: A systematic review and meta-analysis. J. Cardiovasc. Electrophysiol..

[13] Reddy V.Y., Dukkipati S.R., Neuzil P., Natale A., Albenque J.P., Kautzner J., Shah D., Michaud G., Wharton M., Harari D. (2015). Randomized, Controlled Trial of the Safety and Effectiveness of a Contact Force-Sensing Irrigated Catheter for Ablation of Paroxysmal Atrial Fibrillation: Results of the TactiCath Contact Force Ablation Catheter Study for Atrial Fibrillation (TOCCASTAR) Study. Circulation.

[14] Zhou J., Qu F., Sang X., Wang X., Nan R. (2021). Impact of contact force sensing technology on outcome of catheter ablation of idiopathic premature ventricular contractions originating from the outflow tracts. Europace.

[15] Ábrahám P., Ambrus M., Herczeg S., Szegedi N., Nagy K.V., Salló Z., Osztheimer I., Széplaki G., Tahin T., Merkely B. (2021). Similar outcomes with manual contact force ablation catheters and traditional catheters in the treatment of outflow tract premature ventricular complexes. Europace.

[16] Baranowski R., Bieganowska K., Cygankiewicz I., Guzik P., Kurpesa M., Lelonek M., Maciejewska M., Miszczak-Knecht M., Piotrowicz E., Szydło K. (2013). Wytyczne dotyczące wykonywania długotrwałych rejestracji EKG. Stanowisko grupy ekspertów Sekcji Elektrokardiologii Nieinwazyjnej i Telemedycyny Polskiego Towarzystwa Kardiologicznego. Kardiol. Pol..

[17] Steeds R.P., Garbi M., Cardim N., Kasprzak J.D., Sade E., Nihoyannopoulos P., Popescu B.A., Stefanidis A., Cosyns B., Monaghan M. (2017). 2014–2016 EACVI Scientific Documents Committee; 2014–2016 EACVI Scientific Documents Committee. EACVI appropriateness criteria for the use of transthoracic echocardiography in adults: A report of literature and current practice review. Eur. Heart J. Cardiovasc. Imaging.

[18] Zhao Z., Liu X., Gao L., Xi Y., Chen Q., Chang D., Xiao X., Cheng J., Yang Y., Xia Y. (2020). Benefit of contact force–guided catheter ablation for treating premature ventricular contractions. Tex. Heart Inst. J..

[19] Reddy V.Y., Shah D., Kautzner J., Schmidt B., Saoudi N., Herrera C., Jaïs P., Hindricks G., Peichl P., Yulzari A. (2012). The relationship between contact force and clinical outcome during radiofrequency catheter ablation of atrial fibrillation in the Toccata study. Heart Rhythm.

[20] Mizuno H., Vergara P., Maccabelli G., Trevisi N., Eng S.C., Brombin C., Mazzone P., Della Bella P. (2013). Contact force monitoring for cardiac mapping in patients with ventricular tachycardia. J. Cardiovasc. Electrophysiol..

[21] Shah D., Lambert H., Langenkamp A., Vanenkov Y., Leo G., Gentil-Baron P., Walpoth B. (2011). Catheter tip force required for mechanical perforation of porcine cardiac chambers. Europace.

[22] Ikeda A., Nakagawa H., Lambert H., Shah D.C., Fonck E., Yulzari A., Sharma T., Pitha J.V., Lazzara R., Jackman W.M. (2014). Relationship between catheter contact force and radiofrequency lesion size and incidence of steam pop in the beating canine heart: Electrogram amplitude, impedance, and electrode temperature are poor predictors of electrode-tissue contact force and lesion size. Circ. Arrhythm. Electrophysiol..

[23] Schönbauer R., Sommer P., Misfeld M., Dinov B., Fiedler L., Huo Y., Gaspar T., Breithardt O.A., Hindricks G., Arya A. (2013). Relevant ventricular septal defect caused by steam pop during ablation of premature ventricular contraction. Circulation.

[24] Akca F., Janse P., Theuns D.A., Szili-Torok T. (2015). A prospective study on safety of catheter ablation procedures: Contact force guided ablation could reduce the risk of cardiac perforation. Int. J. Cardiol..

[25] Capulzini L., Vergara P., Mugnai G., Salghetti F., Abugattas J.P., El Bouchaibi S., Iacopino S., Sieira J., Enriquez Coutiño H., Ströker E. (2019). Acute and one year outcome of premature ventricular contraction ablation guided by contact force and automated pacemapping software. J. Arrhythm..

[26] Hendriks A.A., Akca F., Dabiri Abkenari L., Khan M., Bhagwandien R., Yap S.C., Wijchers S., Szili-Torok T. (2015). Safety and Clinical Outcome of Catheter Ablation of Ventricular Arrhythmias Using Contact Force Sensing. J. Cardiovasc. Electrophysiol..

[27] Guckel D., Niemann S., Ditzhaus M., Molatta S., Bergau L., Fink T., Sciacca V., El Hamriti M., Imnadze G., Steinhauer P. (2021). Long-Term Efficacy and Impact on Mortality of Remote Magnetic Navigation Guided Catheter Ablation of Ventricular Arrhythmias. J. Clin. Med..

[28] Deutsch K., Ciurzyński M., Śledź J., Zienciuk-Krajka A., Mazij M., Ludwik B., Stec P., Wileczek A., Pruszczyk P., Stec S. (2021). Association between the geographic region and the risk of familial atrioventricular nodal reentrant tachycardia in the Polish population. Pol. Arch. Intern Med..

[29] Fadhle A., Hu M., Wang Y. (2020). The safety and efficacy of zero-fluoroscopy ablation versus conventional ablation in patients with supraventricular tachycardia. Kardiol. Pol..

